# Diagnostic performance of anti-Zika virus IgM, IgAM and IgG ELISAs during co-circulation of Zika, dengue, and chikungunya viruses in Brazil and Venezuela

**DOI:** 10.1371/journal.pntd.0009336

**Published:** 2021-04-19

**Authors:** Ivonne Morales, Kerstin D. Rosenberger, Tereza Magalhaes, Clarice N. L. Morais, Cynthia Braga, Ernesto T. A. Marques, Guilherme Amaral Calvet, Luana Damasceno, Patricia Brasil, Ana Maria Bispo de Filippis, Adriana Tami, Sarah Bethencourt, Mayling Alvarez, Pedro A. Martínez, Maria G. Guzman, Bruno Souza Benevides, Andrea Caprara, Nguyen Than Ha Quyen, Cameron P. Simmons, Bridget Wills, Xavier de Lamballerie, Jan Felix Drexler, Thomas Jaenisch, the IDAMS Clinical Study Group

**Affiliations:** 1 Section Clinical Tropical Medicine, Department for Infectious Diseases, Heidelberg University Hospital, Heidelberg, Germany; 2 German Centre for Infection Research (DZIF), associated partner Heidelberg University Hospital, Heidelberg, Germany; 3 Center for Vector-Borne Infectious Diseases (CVID), Department of Microbiology, Immunology and Pathology, Colorado State University (CSU), Fort Collins, Colorado, United States of America; 4 Laboratory of Virology and Experimental Therapeutics, Aggeu Magalhaes Institute, Oswaldo Cruz Foundation, Recife, Brazil; 5 Department of Parasitology, Aggeu Magalhaes Institute, Oswaldo Cruz Foundation, Recife, Brazil; 6 Institute of Integral Medicine Professor Fernando Figueira (Instituto de Medicina Integral Professor Fernando Figueira-IMIP), Recife, Brazil; 7 Department of Infectious Diseases, Graduate School of Public Health, University of Pittsburgh, Pittsburgh, Pennsylvania, United States of America; 8 Evandro Chagas National Institute of Infectious Diseases, Oswaldo Cruz Foundation, Rio de Janeiro, Brazil; 9 Flavivirus Laboratory, Oswaldo Cruz Institute, Oswaldo Cruz Foundation, Rio de Janeiro, Brazil; 10 University of Groningen, University Medical Center Groningen, Department of Medical Microbiology and Infection Prevention, Groningen, The Netherlands; 11 Facultad de Ciencias de la Salud, Universidad de Carabobo, Valencia, Venezuela; 12 Instituto de Medicina Tropical Pedro Kouri, La Habana, Cuba; 13 Universidade Estadual Do Ceará, Fortaleza, Brazil; 14 Oxford University Clinical Research Unit, Hospital for Tropical Diseases, Ho Chi Minh City, Vietnam; 15 Institute for Vector-Borne Disease, Monash University, Melbourne, Australia; 16 Centre for Tropical Medicine and Global Health, Nuffield Department of Clinical Medicine, Oxford University, Oxford, United Kingdom; 17 Unité des Virus Emergents (UVE Aix Marseille Université, IRD 190, Inserm 1207-IHUMéditerranée Infection), Marseille, France; 18 Institute of Virology, Charité-Universitätsmedizin Berlin, corporate member of Freie Universität Berlin, Humboldt-Universität zu Berlin, Berlin, Germany; 19 Sechenov University, Martsinovsky Institute of Medical Parasitology, Tropical and Vector-Borne Diseases, Moscow, Russia; 20 German Centre for Infection Research (DZIF), associated partner Charité-Universitätsmedizin Berlin, Berlin, Germany; 21 Heidelberg Institute of Global Health (HIGH), Heidelberg University Hospital, Heidelberg, Germany; Oregon Health and Science University, UNITED STATES

## Abstract

**Background:**

Serological diagnosis of Zika virus (ZIKV) infection is challenging because of the antibody cross-reactivity among flaviviruses. At the same time, the role of Nucleic Acid Testing (NAT) is limited by the low proportion of symptomatic infections and the low average viral load. Here, we compared the diagnostic performance of commercially available IgM, IgAM, and IgG ELISAs in sequential samples during the ZIKV and chikungunya (CHIKV) epidemics and co-circulation of dengue virus (DENV) in Brazil and Venezuela.

**Methodology/Principal findings:**

Acute (day of illness 1–5) and follow-up (day of illness ≥ 6) blood samples were collected from nine hundred and seven symptomatic patients enrolled in a prospective multicenter study between June 2012 and August 2016. Acute samples were tested by RT-PCR for ZIKV, DENV, and CHIKV. Acute and follow-up samples were tested for IgM, IgAM, and IgG antibodies to ZIKV using commercially available ELISAs. Among follow-up samples with a RT-PCR confirmed ZIKV infection, anti-ZIKV IgAM sensitivity was 93.5% (43/46), while IgM and IgG exhibited sensitivities of 30.3% (10/33) and 72% (18/25), respectively. An additional 24% (26/109) of ZIKV infections were detected via IgAM seroconversion in ZIKV/DENV/CHIKV RT-PCR negative patients. The specificity of anti-ZIKV IgM was estimated at 93% and that of IgAM at 85%.

**Conclusions/Significance:**

Our findings exemplify the challenges of the assessment of test performance for ZIKV serological tests in the real-world setting, during co-circulation of DENV, ZIKV, and CHIKV. However, we can also demonstrate that the IgAM immunoassay exhibits superior sensitivity to detect ZIKV RT-PCR confirmed infections compared to IgG and IgM immunoassays. The IgAM assay also proves to be promising for detection of anti-ZIKV seroconversions in sequential samples, both in ZIKV PCR-positive as well as PCR-negative patients, making this a candidate assay for serological monitoring of pregnant women in future ZIKV outbreaks.

## Author summary

Zika virus (ZIKV) is transmitted through the bite of infected *Aedes* mosquitos but can also be transmitted sexually or vertically from mother-to-child. The same mosquitoes transmit dengue virus (DENV) and chikungunya virus (CHIKV), which cause similar clinical syndromes. The ZIKV epidemics in the Pacific and the Americas that occurred between 2015 and 2017 were linked to congenital abnormalities, most prominently microcephaly, in newborns. Because most infections are asymptomatic, diagnosis via indirect serological assays is an important strategy. On the other hand, many serological assays are affected by cross-reactivity resulting from prior infections by closely related viruses, such as DENV. This study evaluated three commercially available and widely used immunoassays that detect IgG, IgM or IgA and M (IgAM) antibodies to ZIKV. Our results suggest that the IgAM test performs best by detecting around 90% of RT-PCR confirmed infections. We also detected additional infections that were not detected by RT-PCR. The strength of this study is that it was carried out in two different countries of the American region where several arboviruses are endemic and that sequential blood samples from individual patients were available to evaluate the performance of the tests over time.

## Introduction

Zika virus (ZIKV) is an arbovirus of the family *Flaviviridae* that was isolated in 1947 from a non-human primate in the Zika forest of Uganda [1]. ZIKV is presumed to have spread from Africa to Southeast Asia and circulated quietly for almost six decades [2], until it caused a first local epidemic on Yap island in 2007, followed by epidemics in several Pacific islands, including French Polynesia, New Caledonia, and Easter Island [3–5]. Almost a decade later, ZIKV spread to Latin America and the Caribbean causing a devastating epidemic. Out of 48 countries and territories affected, Brazil alone reported over 200,000 cases by the end of 2016 [6]. Evidence linking ZIKV infection to clusters of microcephaly and neurological disease prompted the World Health Organization (WHO) to declare a Public Health Emergency of International Concern (PHEIC) between February and November 2016 [7–11].

ZIKV is primarily transmitted through the bite of infected *Aedes* mosquitoes, although other modes of transmission, including mother-to-infant, sexual, and via blood transfusions, have also been reported [12–14]. Although the clinical presentation of the disease may include symptoms, such as fever, rash, headache, arthralgia, and conjunctivitis [15], which are characteristic of a dengue virus (DENV) or chikungunya virus (CHIKV) infection [16], recent data by Burger-Calderon *et al*. [17] and Lindsey *et al*. [18], suggest that the clinical spectrum of Zika disease is age-dependent, with children often displaying non-specific clinical manifestations (including rash or fever), while adolescents and adults tend to present with a dengue-like clinical syndrome, including symptoms like arthralgia, arthritis, and myalgia. The challenge of clinical diagnosis is aggravated by the prevalence of asymptomatic infections. Current estimates of the proportion of asymptomatic ZIKV infections vary widely, ranging from ~ 20% to ~ 80%. A meta-analysis found an overall proportion of asymptomatic Zika infections of 61.8%, however the accuracy of this estimate is limited by the high heterogeneity of estimates reported in the literature [19]. Therefore, clinical diagnosis is challenging, especially in settings where more than one arbovirus co-circulates [20,21].

Laboratory identification of ZIKV relies on the detection of viral RNA in body fluids during the acute infection, mostly in blood (serum or plasma) or urine. However, the time window for detection of viral RNA is short and the average level of viral RNA is low [22–24], leading to limited sensitivity of the standard PCR assays [25]. Moreover, the sensitivity of detection varies not only according to the sample type [26–32], but is also subject to variation in diagnostic performance between laboratories in outbreak afflicted areas [33]. Given that most patients only experience mild symptoms or in up to 80% of cases, an asymptomatic infection [3], viral RNA is often undetectable by the time a patient seeks medical care. Hence, serology is potentially relevant as a diagnostic method. Most ZIKV serological tests are based on the detection of IgM or IgG antibodies to envelope or non-structural proteins (NS) [34–37]. Serologic assays based on whole virus antigen can be superior in terms of sensitivity but at the cost of limited specificity [24,37]. Conversely, anti-NS1 antibody detection methods are thought to be more specific while compromising sensitivity [24]. Kinetic studies of antibody production in patients infected with ZIKV have shown that IgM antibodies begin to appear as early as two days of illness onset and may persist for up to 25 months after exposure, followed by the production of long-lasting IgG antibodies a few days later [38–44]. Interestingly, however, a recent study by Henderson *et al*. [45] reported decreasing levels of antibody prevalence to ZIKV in residents of Fiji and French Polynesia two years after the ZIKV outbreak in those locations. Similarly, Moreira-Soto *et al*. [46] observed a decline in seropositivity to ZIKV IgG in residents of Salvador, Northeastern Brazil 1.5–2 years after testing seropositive. These findings suggest that commonly used serological tests may miss a substantial proportion of past ZIKV infections, underscoring the value of samples collected during on-going epidemics.

Although serology has been the cornerstone of diagnostic testing in many resource-poor settings, assay limitations, including suboptimal sensitivity and frequent cross-reactivity with other flaviviruses, constitute a major challenge. Moreover, the degree of antibody cross-reactivity is determined by the history of previous flavivirus infections or vaccinations, which renders the interpretation of results challenging [36]. To circumvent these limitations, plaque reduction neutralization tests (PRNTs) have been included in testing algorithms to confirm positive or equivocal ELISA results [23,24]. Although the PRNT is more specific than commercially available ELISA tests, it may also be affected by cross-reactive neutralizing antibodies; however, the validity of the PRNT increases over time with results from late convalescent samples believed to be more reliable [47,48]. In addition, it is a demanding and time-consuming technique; as such, it is usually only performed in reference laboratories with the required technical expertise [24,34,49].

Additional alternatives for serological testing may include the detection of antibody sub-classes other than IgM and IgG. A report by Zhao *et al*., [42] found similar IgA antibody kinetics to IgM, while two other studies showed that anti-ZIKV IgA provides greater sensitivity than IgM during the acute phase of infection [50,51]. Interestingly, serum IgA antibody titers have been reported to increase more rapidly and to reach higher levels in the context of secondary dengue infections compared to IgM [52,53], suggesting that IgA behaves similarly to IgG.

Few studies have evaluated anti-ZIKV serologic assays using sequential samples from patients living in areas where arboviruses co-circulate. In this study, we assessed the performance of the widely used Euroimmun anti-ZIKV IgG, IgM and IgAM ELISAs, based on NS1, using sequential blood samples collected between 2012 and 2016 from patients with acute undifferentiated febrile illness (or with a history of fever) in Brazil and Venezuela, some of which were confirmed by RT-PCR as DENV, CHIKV, or ZIKV. The period of sample collection occurred during two major arboviral epidemics, the Zika and chikungunya epidemics, and thus, it represents a unique epidemiological time window during which multiple arboviruses, including DENV co-circulated in the Americas.

## Methods

### Ethics statement

Ethical approval for this study was obtained from the Institutional Review Board of the Medical Faculty of Heidelberg University Hospital under protocol S-445/2011 and from local ethics committees of the institutions involved at each site: Aggeu Magalhaes Institute and Instituto de Medicina Integral Professor Fernando Figueira, the National Commission for Ethics in Research (CONEP, Brazil), the Biomedical Research Institute, Carabobo University (Aval Bioetico number CBIIB-[UC]-2013-1) and the Evandro Chagas Institute/Fiocruz (CAAE 15580013.5.2002.5262) in Rio de Janeiro, Brazil. Written consent was provided by each participant or a parent/guardian prior to study enrolment.

### Characteristics of the study population

Between 2012 and 2016, 924 patients in Brazil and Venezuela were enrolled in the IDAMS multicenter observational dengue research study, which was described in detail elsewhere [54]. During this time, the ZIKV epidemic swept through the Americas in the years 2015–16 [55,56], while the CHIKV epidemic moved South from the Caribbean, hitting Venezuela at around 2014, while it peaked in Northern Brazil towards the end of the ZIKV epidemic in 2016 [56,57]. In summary, symptomatic patients ≥ 5 years of age, who presented with fever or a history of fever during the previous 72 hours were included in the study. Out of the 924 patients, 17 patients lacked information regarding day of illness or information that would allow for its estimation and were removed from the analysis. The remaining 907 patients were included in the analysis. In Brazil, 543 (59.9%) patients were enrolled between 2012 and 2016 in four cities representing two different geographical areas: Rio de Janeiro and Resende (n = 109) in the Southeast, and Recife and Fortaleza (n = 434) in the Northeast. The remaining 40.1% of the patients (n = 364) were enrolled in the Venezuelan state of Carabobo between 2013 and 2016. Overall, slightly more females (55.9%) were included in the study than males (44.1%). The age of participants ranged between 5–83 years (mean age 28 years) (Table 1).

**Table 1:** Baseline characteristics of 907 patients from Venezuela and Brazil.

Patient data	Valencia, Venezuela	Rio de Janeiro & Resende, Brazil	Recife & Fortaleza, Brazil	Total
**Number, n**	364	109	434	907
**Gender**				
**female, n (%)**	237 (65.1)	55 (51.9)^a^	213 (49.1)	505 (55.9)^b^
**male, n (%)**	127 (34.9)	51 (48.1)^a^	221 (50.9)	399 (44.1)^b^
**Age in years, mean (SD)**	25.6 (14.8)	31.3 (14.1)^a^	29.3 (13.3)	28.0 (14.2)^b^
**Study period, years**	2013–16	2015–2016 (Rio)	2012–15 (Fortaleza)	2012–16
		2015 (Resende)	2015–2016 (Recife)	
**RT-PCR positives, n positive/n tested (%)**				
**Zika**	30/281 (10.7)	15/106 (14.2)	38/433 (8.8)	83/820 (10.1)
**Dengue/NS1**^**c**^	39/356 (11.0)	22/107 (20.6)	90/433 (20.8)	151/896 (16.9)
**Chikungunya**	121/345 (35.1)	19/109 (17.4)	138/434 (31.8)	278/888 (31.3)

a Calculated from 106 patients since sex and age information were unavailable for 3 patients from Rio de Janeiro.

b Calculated from 904 patients since sex and age information were unavailable for 3 patients from Rio de Janeiro.

c Consists of the number of DENV confirmed infections by RT-PCR or anti-DENV NS1 ELISA. Total number of DENV RT-PCR negative, indeterminate, or not tested but NS1 positive, n = 35. Per site: Rio de Janeiro and Resende; n = 9, Recife and Fortaleza; n = 13, Valencia, n = 13.

At least one and up to three sequential blood samples were collected from participants throughout the course of illness—at enrollment (acute period), at cessation of fever or within six days of enrollment (last acute visit), and 10 or more days after fever onset (which also had to be at least one week after the last acute visit). For the purposes of this study, samples collected between 1 and 5 days of fever onset are referred to as ‘acute samples,’ while those collected beyond 5 days of fever onset are referred to as ‘follow-up samples.’ Blood samples were processed, and plasma was aliquoted and stored at -20° C or -70° C for serologic or molecular testing, respectively. In addition, data on clinical and laboratory parameters were collected and sent to a secure server at Heidelberg University Hospital.

### Laboratory diagnostic assays

#### RT-PCR

Plasma samples were analyzed by real-time PCR (RT-PCR) for the detection of DENV, ZIKV or CHIKV RNA at centralized laboratory locations—at the Aggeu Magalhaes Institute, for Brazilian samples collected in Recife and Fortaleza, at the Oswaldo Cruz Institute, for samples collected in Rio de Janeiro and Resende, or at Institute Pedro Kourí in Havana, Cuba, for samples collected in Valencia, Venezuela. Standardized protocols, primers and reagents were employed to perform RNA extraction and RT-PCR at each location (S1 Table). RNA extraction and procedures for RT-PCR in Recife, Brazil have been described elsewhere [34,58–60]. In brief, virus RNA was extracted using the QIAmp Viral RNA Mini Kit (Qiagen, Valencia, CA, United States) following the manufacturer’s instructions. At the Aggeu Magalhaes Institute in Recife and the Oswaldo Cruz Institute in Rio de Janeiro, RT-PCRs were prepared using the GoTaq Probe 1-Step RT-qPCR System (Promega, Madison, U.S.) and were run using the Applied Biosystems 7500 Real-Time PCR System. At Institute Pedro Kourí, automated RNA purification was conducted in a QIAcube instrument from QIAGEN, Germany, in a final elution volume of 60 microliters. The SuperScript III Platinum One-Step qRT-PCR System was used for RT-PCR amplification using a set of primers described in S1 Table and reactions were run in a Rotor Gene Q (QIAGEN, Germany) cycler. Samples were classified into one of four categories according to their RT-PCR result or the NS1 Platelia Dengue assay (described below): 1) ZIKV RT-PCR positive, 2) DENV RT-PCR/NS1 positive, 3) CHIKV RT-PCR positive or 4) RT-PCR negative if they tested negative to all three viruses and to DENV NS1. The CHIKV PCR status of n = 6 samples from Rio de Janeiro was not available but was inferred as negative since the samples were collected between May and November 2015, before circulation of chikungunya was first detected in Rio de Janeiro.

#### DENV NS1 antigen

To detect DENV NS1 antigen, the Platelia NS1 assay (Biorad, Marnes-la-Coquette, France) was performed on 867 acute (enrollment) samples according to the manufacturer’s instructions.

#### Anti-ZIKV ELISAs

We evaluated the performance of the commercially available anti-ZIKV ELISAs in a total of 832 acute samples and 462 follow-up samples according to the manufacturer’s (EUROIMMUN, Lübeck, Germany) instructions (S2 and S3 Tables). The ZIKV ELISAs were based on the detection of antibodies to the recombinant non-structural protein 1 (NS1). The ratio between the extinction coefficient of the sample and the extinction coefficient of the calibrator were used to define the assays’ reference values. Samples with a ratio value < 0.8 were considered negative, ≥ 0.8 to < 1.1 were labeled as indeterminate, and ≥ 1.1 were considered positive. The limit of detection for the assays was established at a ratio of 1.1.

#### Anti-DENV assays

1,166 acute and 649 follow-up samples were analyzed for the presence of dengue IgM and 1,144 acute and 645 follow-up samples were tested for the presence of dengue IgG using the Panbio IgM or Panbio IgG Capture/Indirect ELISAs (Alere, Waltham, U.S.), respectively, according to the manufacturer’s instructions.

#### Statistics

Statistical analyses were performed using the R statistical program (R Foundation for Statistical Computing, Vienna, Austria) version 3.6.1. Sensitivity for IgM, IgAM, and IgG (acute or follow-up tests) was calculated as the proportion of positive results for each assay among RT-PCR confirmed ZIKV positive samples. Specificity was calculated as the proportion of samples that tested negative for anti-ZIKV antibodies among individuals with a DENV infection confirmed by RT-PCR or DENV NS1, assuming a low probability of dual infection. However, we are aware that antibody reactivity could result from a previous infection, i.e. an acute infection 6 months ago and thus not represent a false positive result in the strict sense. In addition, samples with indeterminate serological results or co-infections were excluded from sensitivity (except for one acute and one follow-up sample from one patient that tested positive by IgAM only in the follow-up sample and who was RT-PCR positive for ZIKV but with indeterminate results in anti-DENV NS1), specificity, and seroconversion determination. The associations between potential co-factors (fever, skin flush, skin rash, testing dengue IgG positive at enrollment, study site, year, age, sex, and RT-PCR result) and anti-ZIKV IgG reactivity at enrollment were assessed with logistic regressions using the glmuni and glmmulti functions of the ‘finalfit’ package in R. Stepwise selection based on minimization of the Akaike Information Criterion (AIC) was implemented on the adjusted model using the step function in R to identify the predictor variables in the model resulting in the lowest AIC. Participants with indeterminate results for ZIKV IgG at enrollment, DENV IgG at enrollment, ZIKV RT-PCR, CHIKV RT-PCR, RT-PCR Neg as well as those participants with indeterminate results for both DENV RT-PCR and NS1 were excluded from regression models.

## Results

Among the 907 patients included in this study, 820 had an acute sample tested by RT-PCR for ZIKV. Out of these, 83 (10.1%) were confirmed as ZIKV positive and six were co-infections with DENV (n = 4) or CHIKV (n = 2). From a total of 896 patients, 151 (16.9%) tested positive by RT-PCR to DENV or by the DENV NS1 ELISA, and 278/888 (31.3%) patients were confirmed as CHIKV infected by RT-PCR (Table 1).

### Sensitivity of anti-ZIKV ELISAs in acute and follow-up samples

We evaluated the sensitivity of the commercially available NS1-based IgM, IgAM, and IgG ELISAs in a panel of acute (illness day 1–5) and follow-up (illness day ≥ 6) samples from 77 patients with RT-PCR confirmed ZIKV infection (Table 2). The number of acute and follow-up samples analyzed with each antibody ELISA test varied. The sensitivity of the IgM ELISA during acute timepoints of illness was low—9.4% (95% Confidence Interval [CI]: 3.9–20.0%)—, increasing to 30.3% (CI: 16.2–48.9%) among follow-up samples, while the IgAM ELISA yielded sensitivities of 15.5% (CI: 8.4–26.5%) and 93.5% (CI: 81.1–98.3%) in acute and follow-up samples, respectively. When comparing samples tested with both IgM and IgAM, results were similar (S4 Table) to those presented in Table 2. The sensitivity of the IgG ELISA was found to be 41.4% (CI: 30.0–53.8%) in acute samples and 72.0% (CI: 50.4–87.1%) in follow-up samples.

**Table 2:** Sensitivity values of anti-ZIKV IgM, IgAM, and IgG ELISAs.

Serological test	ZIKV RT-PCR+ Number of patients = 77									
	Acute (day 1–5)					Follow-up (≥ 6 day ≤ 31)				
	N	Pos	Neg	Ind	% Sensitivity (95% CI)	N	Pos	Neg	Ind	% Sensitivity (95% CI)
**IgM**	66	6	58	2	9.4 (3.9–20.0)	35	10	23	2	30.3 (16.2–48.9)
**IgAM**	76	11	60	5	15.5 (8.4–26.5)	48	43	3	2	93.5 (81.1–98.3)
**IgG**	76	29	41	6	41.4 (30.0–53.8)	25	18	7	0	72.0 (50.4–87.1)

N represents the number of patients with a sample that was tested for the corresponding immunoassay; some patients have both acute and follow-up samples: IgM, n = 34; IgAM, n = 47; IgG, n = 24.

**Fig 1:**
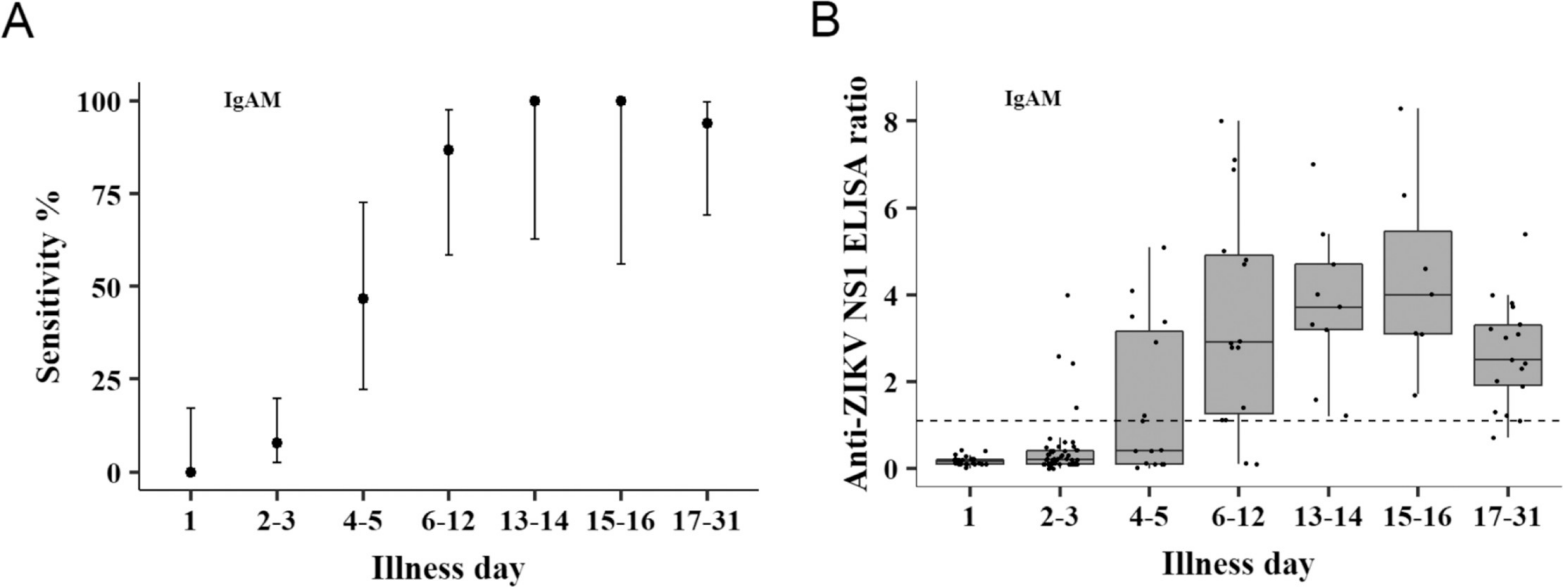
Anti-ZIKV IgAM sensitivity and ratio values by illness day in ZIKV positive patients. (A) Sensitivity of IgAM to ZIKV per illness day. IgAM immunoassay results from 140 samples collected between day 1 and 31 from ZIKV positive patients were used to calculate assay sensitivity at different illness days. The number of tests corresponding to each day or day range are the following: day 1, n = 24; days 2–3, n = 51; days 4–5, n = 15; days 6–12, n = 15; days 13–14, n = 9; days 15–16, n = 7 and days 17–31, n = 17. Bars represent the 95% confidence intervals. (B) Box plot of anti-ZIKV IgAM ratio values per illness day. The number of tests corresponding to each day or day range are the same as in (A). The dashed horizontal line indicates the assay’s cut-off ratio value for positive results set at 1.1.

Fig 1 illustrates the sensitivity of the IgAM ELISA in ZIKV RT-PCR positive samples by illness day (Fig 1A) and corresponding IgAM antibody ratio values (Fig 1B). Out of 140 acute and follow-up samples tested, 92 (65.7%) were collected between days 1 and 5 and 48 (34.3%) between days 6 and 31. At days 4–5, 7/15 samples tested positive, resulting in a sensitivity of 46.7%. At 6–12 days of illness, sensitivity increased to 86.7% (13/15) and reached 100% (9/9) at days 13–14 of illness and remained at 100% (7/7) at days 15–16. Between days 17 and 31, sensitivity moderately decreased to 94.1% (16/17) (Fig 1A). The median IgAM antibody ratio level at 4–5 days of illness was 0.4 (range, 0–5.1), which is below the cut-off ratio of the immunoassay. In contrast, beyond day 6, the median IgAM ratio levels were above the cut-off: 2.9 (range, 0.1–8), 3.7 (range, 1.2–7) and 4.0 (range, 1.7–8.3) at 6–12, 13–14, and 15–16 days of illness, respectively. The median IgAM ratio level decreased between 17 and 31 days of illness to 2.5 (range, 0.7–5.4). In comparison, the sensitivity of IgM and IgG at days 13–14 were 50% (3/6) and 100% (3/3) and 50% (3/6) and 40% (2/5) at days 15–16, respectively (S1A and S1C Fig). In addition, IgG sensitivity did not show a gradual increase over time, but this may be due to the low number of samples tested at the given day of illness ranges depicted in the graph. The corresponding median IgM or IgG antibody ratio levels at 13–14 and 15–16 days of illness were 0.7 (range, 0.1–3.2) and 1.0 (range, 0.2–4.7) for IgM and 3.5 (range, 1.5–3.5) and 0.3 (range, 0–3.1) for IgG (S1B and S1D Fig), all of which are below the observed median antibody ratio values for IgAM at the same illness days (Fig 1B).

### Anti-ZIKV seroconversions in samples with a confirmed ZIKV infection

We tested for anti-ZIKV IgM, IgAM, and IgG seroconversions in paired samples from 55 ZIKV positive patients (Table 3). Seroconversions for IgM and IgAM were detected in 12/42 (28.6%) and 42/51 (82.4%) patients, respectively. Out of 38 paired samples tested with both IgM and IgAM, 11 seroconversions were detected by both immunoassays. IgAM detected an additional 19 seroconversions that were not detected with IgM. In samples without a seroconversion for IgAM, we did not detect a seroconversion for IgM at any timepoint. IgG seroconversions were detected in 10/22 (45.5%) patients (Table 3). Out of the 10 patients with a seroconversion for IgG, 100% (10/10) and 90% (9/10) were IgAM or IgM positive, respectively. Overall, seroconversion for ZIKV-specific IgM, IgAM, or IgG antibodies could be expected to occur between 2–31 days of illness (S2 Fig).

**Table 3:** Seroconversions for anti-ZIKV IgM, IgAM, and IgG in patients with paired sera.

RT-PCR+ (n)^a^	Seroconversion % (Number of positive patients in a 2^nd^ sample/Number of negative patients in the 1^st^ sample)		
	IgM	IgAM	IgG
**ZIKV (55)**	12/42 (28.6%)	42/51 (82.4%)	10/22 (45.5%)
**DENV/NS1 (105)**	3/76 (3.9%)	9/96 (9.4%)	22/66 (33.3%)
**CHIKV (101)**	0/22 (0.0%)	2/97 (2.1%)	0/32 (0.0%)
**RT-PCR Negative (238)**^**b**^	7/168 (4.2%)	26/220 (11.8%)	10/91 (11.0%)

Seroconversions were tested in paired sera, where the first sample of the pair tested negative for the respective serological test. The time period of sample collection and testing corresponds to illness days 1–31.

a Shows the number of patients that were positive to one of the three viruses by RT-PCR (or positive for DENV by DENV NS1) or negative to all three viruses and who had sequential samples available to test for seroconversions.

b Consists of patients that tested negative to all DENV/NS1, ZIKV, and CHIKV.

### Anti-ZIKV seroconversions in RT-PCR negative samples

We next investigated seroconversions for anti-ZIKV-specific IgM, IgAM, and IgG antibodies among 238 samples that tested negative to all three viruses by RT-PCR. Seroconversions for ZIKV-specific IgM, IgAM, and IgG antibodies were detected in 7/168 (4.2%), 26/220 (11.8%), and 10/91 (11.0%) paired samples, respectively (Table 3). In paired samples (n = 152) tested with both the IgM and IgAM immunoassays, 7 seroconversions were detected by both. IgAM detected 10 additional seroconversions that were not detected by IgM. All seroconversions for IgM were detected by the IgAM ELISA. From the RT-PCR negative samples to all three viruses, 85 paired samples were tested with all three ELISAs, of which IgM, IgAM, and IgG detected 5 (5.9%), 9 (10.6%), and 8 (9.4%) seroconversions, respectively.

### Anti-ZIKV seroconversions in DENV/NS1 or CHIKV positive samples

Seroconversions for ZIKV-specific IgM, IgAM, and IgG antibodies were also observed in DENV RT-PCR positive/NS1 positive samples (DENV/NS1 positive). Seroconversions for anti-ZIKV IgAM were detected in 9/96 (9.4%), while IgM seroconversions were only detected in 3/76 (3.9%) DENV/NS1 positive samples (Table 3). Seroconversion for anti-ZIKV IgG was detected in 22/66 (33.3%) DENV/NS1 positive samples (Table 3). Of these 22 seroconversions, only one was anti-ZIKV IgM positive and five were anti-ZIKV IgAM positive.

A total of 59 DENV/NS1 positive samples were tested by all three immunoassays (IgM, IgAM, IgG). Out of these, IgM, IgAM, and IgG detected 1 (1.7%), 5 (8.5%) and 21 (35.6%) seroconversions, respectively. All IgM and IgAM seroconversions were also detected by IgG.

In CHIKV RT-PCR positive samples, seroconversions for anti-ZIKV IgAM were detected in 2/97 (2.1%) samples tested, while no seroconversions for either IgM or IgG were detected (Table 3).

### Potential cross-reactivity and assessment of specificity

To explore the possibility that the anti-ZIKV antibody seroconversions observed in DENV RT-PCR/NS1 positive samples could be due to cross-reactivity with anti-DENV antibodies, we examined anti-DENV IgM reactivity in samples where a seroconversion for anti-ZIKV IgM or IgAM was detected. All nine seroconversions for anti-ZIKV IgAM were also positive for anti-DENV IgM. Six out of the nine seroconversions for anti-ZIKV IgAM also seroconverted for anti-DENV IgM, while 2/3 seroconversions for ZIKV IgM were DENV IgM positive (one due to a seroconversion for DENV IgM). In the case of anti-ZIKV IgG, all 22 anti-ZIKV IgG seroconversions were also positive for anti-DENV IgG antibodies (15 were due to seroconversions for DENV IgG).

In regions endemic for several flaviviruses, cross-reacting antibodies from prior infections may affect the validity of the anti-ZIKV immunoassays. Using multiple regression analysis, we analyzed factors that were associated with anti-ZIKV IgG reactivity at baseline during the acute phase of the illness (S5 Table). The combination of a past dengue infection, an acute CHIKV infection, and being 16 years of age or older were among the factors linked to having anti-ZIKV IgG antibodies at baseline.

Specificity is challenging to assess as antibody reactivity can be attributed to cross-reactivity or to prior infection and a persistent immune response. The specificity of the anti-ZIKV IgM and IgAM test results was calculated using a panel of samples (n = 136) that tested negative to ZIKV by RT-PCR, but that were positive for DENV by RT-PCR/NS1, assuming that the probability for dual infection was low (Table 4).

**Table 4:** Anti-ZIKV IgM and IgAM ELISA specificity.

Serological test	DENV RT-PCR+ / NS1+ Number of patients = 136									
	Acute (day 1–5)					Follow-up (≥ 6 day ≤ 31)				
	N	Pos	Neg	Ind	% Specificity (95% CI)	N	Pos	Neg	Ind	% Specificity (95% CI)
**IgM**	92	2	89	1	97.8 (91.5–99.6)	57	4	52	1	92.9 (81.9–97.7)
**IgAM**	134	10	118	6	92.2 (85.7–96.0)	86	12	68	6	85.0 (74.9–91.7)

N represents the number of patients with samples; some patients have both acute and follow-up samples: IgM, n = 56; IgAM, n = 86.

Anti-ZIKV IgM specificity in DENV/NS1 positive samples amounted to 97.8% (CI: 91.5–99.6%) in acute samples and 92.9% (CI: 81.9–97.7%) in follow-up samples. The specificity of the anti-ZIKV IgAM ELISA in acute samples positive for DENV/NS1 was 92.2% (CI: 85.7–96.0%) in acute and 85.0% (CI: 74.9–91.7%) in follow-up samples. When specificity calculation was limited to samples tested with both IgM and IgAM immunoassays, the specificities detected with IgM remained the same, while a slight decrease in specificity was detected with the IgAM ELISA in the follow-up samples with 81.5% (CI: 68.1–90.3%) (S6 Table), compared to 85.0% in Table 4.

## Discussion

The development of sensitive and specific diagnostic tests that are able to distinguish between the antigenically related flaviviruses is a priority, not only to provide patients with an accurate diagnosis, but also to provide timely and adequate clinical care, especially in the context of ZIKV infections. In the present study, we evaluated the diagnostic performance of the commercially available and widely used IgM, IgAM, and IgG immunoassays based on the ZIKV NS1 protein in a cohort of symptomatic patients with available RT-PCR confirmation on ZIKV, DENV, and CHIKV, living in flavivirus endemic settings between June 2012 and August 2016, a time period that coincided with the Zika and chikungunya epidemics in the Americas.

In the samples collected between 6 and 31 days of illness, IgM sensitivity was estimated at 30.3%. This finding is in stark contrast to results from Steinhagen *et al*. [49], who reported an IgM sensitivity of up to 100% in samples from five travelers to ZIKV endemic regions, while our study assessed 35 samples from residents of flavivirus endemic settings. Interestingly, the same study reported sensitivities in the same range as our study for residents from endemic countries, namely 31.6% and 41.7% for samples collected ≥ 1 day post-illness onset (dpio) and ≥ 6 dpio, respectively. High sensitivity in travelers was also observed in a study by Lustig *et al*. [41], where IgM sensitivity was 79% in specimens collected from ZIKV RT-PCR and/or neutralization positive travelers within the first month after illness onset. Interestingly, decreased IgM sensitivity has been reported in residents of endemic areas in whom a secondary flavivirus infection is more likely than in travelers [41,49], which could potentially be due to low or undetectable IgM levels in secondary flavivirus infections [41,61]. In line with our findings, studies by other authors have shown IgM sensitivities below 60% in ZIKV RT-PCR confirmed samples collected at timepoints under 30 days after onset from patients in endemic regions, including Brazil, Guadeloupe, and French Guiana [51,62–64]. In acute samples collected and tested between day 1–5 of illness, we found a low sensitivity (9.4%) for the IgM ELISA. Similarly, studies by Kadkhoda *et al*. [65] and Kikuti *et al*. [62] observed no IgM positive samples up to five days after illness onset or in acute samples with a median time between illness onset and sample collection of 2.5 days, respectively.

Recently, Bozza *et al*. [50], reported a two-fold increase in sensitivity of an NS1-based IgA immunoassay over IgM and the authors suggested its use as an alternative or complementary marker to IgM to detect acute ZIKV infection. The utility of IgA as a diagnostic marker has been shown for various viral diseases, including dengue [52,53]. Consistent with these reports, we found that the combined IgAM immunoassay displayed increased sensitivity over the IgM immunoassay in ZIKV RT-PCR positive patients. IgAM sensitivity reached 93.5% in samples collected between days 6 and 31. The highest median IgAM antibody ratio value was observed between day 15 to 16 (median antibody ratio = 4.0, range, 1.7–8.3). On days 13–14 and 15–16, all IgAM antibody ratio values were above the cut-off level. Overall, antibodies were detectable from day 2 onwards. In line with these observations, a report by Zhao *et al*. [42], found IgA in serum and saliva from two ZIKV infected travelers to peak around days 10–12 after disease onset and decline thereafter. Similarly, Warnecke *et al*. [51], reported a 93.5% sensitivity of IgA in RT-PCR confirmed ZIKV samples from Dominican Republic residents collected at 8 to 16 days after illness onset. When they combined results from the IgA assay with IgM, sensitivity increased to 100%. In our study, a total of five follow-up samples (day of illness range, 8–23) from patients that were initially ZIKV RT-PCR positive, tested negative for IgAM or had indeterminate results. The difference between the IgA/M sensitivity reported by Warnecke *et al*. [51] and this study may be due to the differential sensitivity of the combined IgAM assay versus running the independent assays. In addition, unlike Warnecke *et al*., we did not consider indeterminate or borderline results into the calculation of sensitivity. Alternatively, IgA class switching may not have occurred yet in these patients [52].

The sensitivity of the IgG immunoassay corresponded to 72.0% in samples collected between days 6 and 31 (and 41.4% in samples collected between days 1 and 5). Other groups have reported sensitivities in the range of 37.5–100% in ZIKV positive samples collected after 1 dpso from travelers or residents of endemic areas [49,51,62–64]. The wide range of sensitivities displayed by IgG reflects the complexity of antibody kinetics in populations with or without prior history of exposure to dengue or other flaviviruses.

Molecular detection of ZIKV RNA using RT-PCR is affected by low viral loads and by the variability of its persistence in different body fluids [25]. Thus, in this context, detection of seroconversion for ZIKV IgM/A antibodies is useful to identify an infection that might have been missed by PCR. The interpretation of IgG seroconversions is dependent on the endemicity of co-circulating flaviviruses.

In this study, serological assays were able to detect incident ZIKV infections by anti-ZIKV antibody seroconversion that were not detected by RT-PCR. Twenty-six seroconversions were detected by the anti-ZIKV IgAM immunoassay, contributing an additional 24% (26/109) of incident ZIKV infections compared to those identified by RT-PCR alone, n = 83.

Our findings also suggest a substantially higher estimate of the frequency of dual infections than previously identified by RT-PCR alone (n = 6). Assuming that at least the IgAM seroconversions represent true ZIKV infections, 9 potential co-infections of ZIKV/DENV were detected in DENV RT-PCR/NS1 positive samples, while two potential ZIKV/CHIKV co-infections were detected in CHIKV RT-PCR-positive samples, contributing an additional ~65% (11/17) of co-infections.

While we cannot rule out with certainty that anti-ZIKV antibody seroconversions were due to cross-reactivity by DENV infections, we can gather additional evidence from the period of sample collection. Although in DENV RT-PCR/NS1 positive samples, six out of nine seroconversions for IgAM were seroconversions for anti-DENV IgM antibodies as well, it is worth noting that these samples were collected during the peak of the Zika epidemic in 2015/16. In contrast, samples that seroconverted for IgG (n = 22) were collected in Fortaleza, Northeastern Brazil, between 2012 and 2015, a period that coincided with two dengue epidemics. In addition, only 5 of the seroconversions detected by IgG in DENV RT-PCR/NS1 positive samples were anti-ZIKV IgAM or IgM positive.

While these findings need to be interpreted with caution due to the potential of false-positive results as a consequence of antibody cross-reactivity to other endemic flaviviruses, we suggest that seroconversions represent good evidence of true ZIKV infection, especially by IgAM.

Our regression analysis suggested that potential sources of antibody cross-reactivity, at least during the acute phase of a ZIKV infection, may be associated to older age and a past or present arboviral infection (S5 Table).

Specificity is challenging to assess in a situation of high endemicity for several arboviruses due to the persistence of antibodies and the antigenic similarities between flaviviruses. Acknowledging the uncertainties around the concept of specificity in this context, we estimated high specificities of 97.8% and 92.9% for the ZIKV IgM ELISA among dengue confirmed infections 1–5 and 6–31 dpso, respectively. Our estimates for specificity agree with the previously published literature [51,62,63,66–68] where IgM specificities in the range of 89.4–100% were reported. Specificities for the IgAM immunoassay were estimated at 92.2% and 85.0% for 1–5 and 6–31 dpso, respectively. Warnecke *et al*. reported a total IgA specificity of 95.0% in DENV positive samples collected 1–19 dpso, which decreased to 91.7% in samples positive for DENV IgA antibodies and up to 83.3% in samples collected 17–19 dpso [51]. Another study by Mendoza *et al*. reported an IgAM specificity of 100% in samples confirmed ZIKV negative by PRNT [68]. However, the samples evaluated in that study were collected from Canadian individuals with travel history to ZIKV endemic areas. Hence, our findings may be more reflective of the antibody cross-reactivity patterns in regions endemic for flaviviruses.

A limitation of this study was the use of samples from symptomatic patients only. The strengths of this study include the use of clinically well-characterized, paired patient samples collected prospectively over the course of the illness and confirmed for ZIKV infection by RT-PCR. The availability of samples with a molecular diagnosis for ZIKV, DENV, and CHIKV infections allowed us to have a reliable standard to investigate assay validity. Moreover, since recruitment for this study coincided with the arrival of the Zika epidemic in the Americas, we were able to test the performance of three commercially available immunoassays during an outbreak scenario in regions where prior infections with one or more flavivirus are common, including different DENV serotypes; thus, results are representative of the complex immunological landscape in flavivirus endemic settings.

In conclusion, this study confirms that IgA antibodies are a useful marker of ZIKV infection. With an estimated sensitivity of ~ 90%, the commercially available IgAM immunoassay outperformed IgM and IgG after day 6 of illness. Moreover, seroconversion for anti-ZIKV IgAM led to the detection of an additional 24% of ZIKV infections and ~65% of co-infections with DENV or CHIKV. This verifies the utility of IgAM as a diagnostic marker of ZIKV infection in regions with co-circulation of multiple arboviruses. These findings are applicable to research settings and serosurveillance of pregnant women, where sequential samples may be collected for detection of seroconversions at timepoints where PCR can no longer detect the virus.

## Supporting information

S1 Table Description of real-time PCR (RT-PCR) primer/probe sets for Zika virus (ZIKV), dengue virus (DENV) and chikungunya virus (CHIKV). Fwd, Forward; Rev Reverse.
(DOCX)

S2 Table Number of samples tested with ZIKV serological assays, stratified by RT-PCR confirmation for ZIKV, DENV, CHIKV, Neg, and by location. ^a^ Day of illness 1–5, shows the total number of acute samples tested per ELISA. Some patients have more than one acute sample tested (Table in S3 Table). ^b^ Day of illness 6 and over, shows the total number of follow-up samples tested per ELISA. Some patients have more than one follow-up sample tested (Table in S3 Table). ^c^ Number of acute samples that tested positive to the respective virus by RT-PCR and tested negative to all other viruses, thus co-infections are excluded. Neg = samples that tested negative to all three viruses and to DENV NS1. ^d^ Consists of samples positive for DENV by RT-PCR or samples positive by the DENV NS1 assay. Those positive by NS1 were either negative, indeterminate or not tested for DENV by RT-PCR. The number of samples NS1 positive per site were as follows: Rio de Janeiro and Resende, n = 9; Fortaleza and Recife, n = 8; Valencia, n = 12. ^e^ Includes one sample that tested DENV negative by RT-PCR but with an indeterminate result for DENV NS1. ^f^ Represents the number of total unique acute samples serologically tested per RT-PCR result and per study site. ^g^ Represents the number of total unique follow-up samples serologically tested per RT-PCR result and per study site.
(DOCX)

S3 Table Number of patients per study site that had more than one acute or follow-up sample collected and tested for IgM, IgAM, or IgG antibodies. ^a^ Brazil: Recife, Fortaleza, Rio de Janeiro, and Resende; Venezuela: Valencia. Neg = samples that tested negative to all ZIKV, DENV, CHIKV by RT-PCR and negative to DENV NS1 antigen by the Platelia NS1 assay. NA, not applicable. Indicates that no samples were tested for the respective ELISA.
(DOCX)

S4 Table Sensitivity of anti-ZIKV IgM and IgAM ELISAs in samples tested with both serological assays. N represents the number of patients with a sample that was tested for the corresponding immunoassay; some patients have both acute and follow-up samples: IgM, n = 34; IgAM, n = 34.
(DOCX)

S5 Table Univariable, multivariable logistic, and stepwise regression analysis on ZIKV IgG positivity at study enrollment. OR, Odds ratio; CI, confidence interval. The table shows the OR and the respective 95% CIs for the association between testing anti-ZIKV IgG positive and key factors at study enrollment. Results are shown for the univariable and multivariable models, followed by a stepwise regression analysis. Prior to adjustment, we found statistically significant associations between testing anti-ZIKV IgG positive and having fever, being anti-DENV IgG positive at enrollment, being CHIKV RT-PCR positive, being DENV RT-PCR positive or NS1 positive, year, age, and study site (Recife and Valencia compared to Rio de Janeiro/Resende). In the multivariable model, the magnitude of the associations for age, year, and being DENV RT-PCR positive/NS1 positive slightly decreased but remained statistically significant. In contrast, the magnitude of the associations for fever, testing DENV IgG positive at enrollment, and being CHIKV RT-PCR positive increased and remained statistically significant for all. Study site was excluded from the multivariable regression analysis due to collinearity with the variable ‘DENV IgG+.’ Stepwise regression analysis revealed that being DENV IgG positive at enrollment and being CHIKV RT-PCR positive significantly increased the odds of testing ZIKV IgG positive at enrollment by 5.6- and 3.3-fold, respectively. This may be the result of antibody cross-reactivity—in the case of dengue—or due to the overlapping arbovirus epidemics that preceded (CHIKV before ZIKV in Venezuela) or followed the ZIKV epidemic (CHIKV following the ZIKV epidemic in Brazil) (S3 Fig). Having fever decreased the odds (OR = 0.51, CI: 0.33–0.77, p = 0.002) of testing ZIKV IgG positive at enrollment, likely because fever is a marker of acute infection and not past flavivirus exposure. We also found that having tested DENV RT-PCR positive/NS1 positive significantly decreased the odds (OR = 0.32, CI: 0.15–0.62, p = 0.001) of testing ZIKV IgG positive. Being ZIKV IgG positive was significantly more likely among individuals aged 16 years or older by 2.0-fold. This finding may be the result of having a higher proportion of study participants above the age of 16; alternatively, it may be due to cross-reactive antibodies to DENV IgG, which would be expected to be more prevalent among the older age groups. Finally, the years 2015 and 2016 were significantly associated with testing ZIKV IgG positive by 1.9-fold compared to the study period between 2012 and 2014. This observation may be attributed to the peak of the ZIKV epidemic during 2015–16.
(DOCX)

S6 Table Specificity of anti-ZIKV IgM and IgAM ELISAs in samples tested with both serological assays. N represents the number of patients with samples; some patients have both acute and follow-up samples: IgM, n = 56; IgAM, n = 56.
(DOCX)

S1 Fig Anti-ZIKV IgM and IgG sensitivity and ratio values by illness day in ZIKV positive patients. (A) Sensitivity of IgM to ZIKV per illness day. IgM immunoassay results from 108 samples collected between day 1 and 31 from ZIKV positive patients were used to calculate assay sensitivity at different illness days. The number of tests corresponding to each day or day range are the following: day 1, n = 17; days 2–3, n = 44; days 4–5, n = 13; days 6–12, n = 11; days 13–14, n = 6; days 15–16, n = 6 and days 17–31, n = 11. Bars represent the 95% confidence intervals. (B) Box plot of anti-ZIKV IgM ratio values per illness day. The number of tests corresponding to each day or day range are the same as in (A). The dashed horizontal line indicates the assay’s cut-off ratio value for positive results set at 1.1. (C) Sensitivity of IgG to ZIKV per illness day. IgG immunoassay results from 104 samples collected between day 1 and 31 from ZIKV positive patients were used to calculate assay sensitivity at different illness days. The number of tests corresponding to each day or day range are the following: day 1, n = 24; days 2–3, n = 46; days 4–5, n = 9; days 6–12, n = 11; days 13–14, n = 3; days 15–16, n = 5 and days 17–31, n = 6. Bars represent the 95% confidence intervals. (D) Box plot of anti-ZIKV IgG ratio values per illness day. The number of tests corresponding to each day or day range differ slightly from (C) since some antibody reference values were not available: day 1, n = 21; days 2–3, n = 41; days 4–5, n = 8; days 6–12, n = 10; days 13–14, n = 3; days 15–16, n = 5 and days 17–31, n = 6. The dashed horizontal line indicates the assay’s cut-off ratio value for positive results set at 1.1.
(TIF)

S2 Fig Timeframe for seroconversion detection for IgM, IgAM, and IgG antibodies in Zika virus (ZIKV) positive patients. IgM (red line), IgAM (blue line), IgG (green line). The timeframe for seroconversion detection is shown for each patient that had an initial sample that tested negative to one or more of the antibody ELISAs and who seroconverted at a later timepoint. The colored dots closest to the x-axis indicate the illness day at which the first sample was collected and tested negative for each immunoassay. The second colored dot from bottom to top, indicates the first illness day at which seroconversion could have occurred, which corresponds to one day after the first sample tested negative. The topmost colored dot indicates the illness day at seroconversion detection. Black dots indicate the median illness day.
(TIF)

S3 Fig Epidemiological curves of Zika virus (ZIKV), dengue virus (DENV), and chikungunya virus (CHIKV) cases confirmed by RT-PCR from 2012–2016 in Brazil and Venezuela. ZIKV (gray line), DENV (blue line), CHIKV (red line). Epidemiological curves are shown for Fortaleza and Recife in Northeastern Brazil, Rio de Janeiro and Resende in Southeastern Brazil, and Valencia in Northern Venezuela.
(TIF)
