# Supplementary material for: Diagnostic performance of anti-Zika virus IgM, IgAM and IgG ELISAs during co-circulation of Zika, dengue, and chikungunya viruses in Brazil and Venezuela
Source: PLoS Negl Trop Dis. 2021 Apr 19;15(4):e0009336. doi: 10.1371/journal.pntd.0009336 (PMC8084345; doi:10.1371/journal.pntd.0009336)
Supplement: S1 Table — Fwd, Forward; Rev Reverse. (DOCX) [file pntd.0009336.s001.docx]

| Site | Primer | Sequence (5‘-3‘) | References |
| --- | --- | --- | --- |
| Rio de Janeiro & Resende | ZIKV Fwd | CCGCTGCCCAACACAAG |  |
|  | ZIKV Rev | CCACTAACGTTCTTTTGCAGACAT | [1] |
|  | ZIKV Probe | **FAM**-AGCCTACCTTGACAAGCAGTCAGACACTCAA-**BHQ1** |  |
|  | DENV-G Fwd | as described in [2] |  |
|  | DENV-G Rev | as described in [2] | [2] |
|  | DENV-G Probe | as described in [2] |  |
|  | CHIKV Fwd | TCACTCCCTGTTGGACTTGATAGA |  |
|  | CHIKV Rev | TTGACGAACAGAGTTAGGAACATACC | [3] |
|  | CHIKV Probe | **FAM**-AGGTACGCGCTTCAAGTTCGGCG-**BHQ1** |  |
| Recife & Fortaleza |  |  |  |
|  | ZIKV Fwd | CCGCTGCCCAACACAAG |  |
|  | ZIKV Rev | CCACTAACGTTCTTTTGCAGACAT | [1] |
|  | ZIKV Probe | **VIC**-AGCCTACCTTGACAAGCAGTCAGACACTCAA-**BHQ1** |  |
|  | DENV-G Fwd | AAGGACTAGAGGTTAGAGGAGACCC |  |
|  | DENV-G Rev | CGTTCTGTGCCTGGAATGATG | [4, 5] |
|  | DENV-G Probe | **FAM**-AACAGCATATTGACGCTGGGAGAGACCAGA-**BHQ1** |  |
|  | CHIKV Fwd | TCACTCCCTGTTGGACTTGATAGA |  |
|  | CHIKV Rev | TTGACGAACAGAGTTAGGAACATACC | [3] |
|  | CHIKV Probe | **FAM**-AGGTACGCGCTTCAAGTTCGGCG-**BHQ1** |  |
| Valencia |  |  |  |
|  | ZIKV Fwd | as described in [1, 6] |  |
|  | ZIKV Rev | as described in [1, 6] | [1, 6] |
|  | ZIKV Probe | as described in [1, 6] |  |
|  | DENV-1 Fwd | ATCCATGCCCAYCAYCAAT |  |
|  | DENV-1 Rev | YRTGRGTTTTRTCCTCCATC |  |
|  | DENV-1 Probe | **FAM**-TCAGTGTGGAATAGGGTTTGGATAGAGGAA-**BHQ1** |  |
|  | DENV-2 Fwd | TCCATACAYGCYAARCATGAA |  |
|  | DENV-2 Rev | GGGAKTTCMTCCCATGATTCC |  |
|  | DENV-2 Probe | **JOE**-AGGGTGTGGAT**Y**C**R**AGAAAACCC**R**TGG-**BHQ1** |  |
|  | DENV-3 Fwd | TTTCTGCTCCCACCACTTTC |  |
|  | DENV-3 Rev | CCATCCYGCTCCTTGAGA |  |
|  | DENV-3 Probe | **TEXAS RED**-AAGAAAGTTGGTAGTTCCCTGCAGACCCCA-**BHQ1** |  |
|  | DENV-4 Fwd | GYGTGGTGAAGCCYCTRGAT |  |
|  | DENV-4 Rev | AGTGARCGGCCATCCTTCAT |  |
|  | DENV-4 Probe | **CY5**-ACTTCCCTCCTCTTYTTGAACGACATGGGA-**BHQ1** |  |
|  | CHIKV Fwd | as described in [6] |  |
|  | CHIKV Rev | as described in [6] | [6] |
|  | CHIKV Probe | as described in [6] |  |

## **References**

1. Lanciotti RS, Kosoy OL, Laven JJ, Velez JO, Lambert AJ, Johnson AJ, et al. Genetic and serologic properties of Zika virus associated with an epidemic, Yap State, Micronesia, 2007. Emerg Infect Dis. 2008;14(8):1232-9.

2. Santiago GA, Vergne E, Quiles Y, Cosme J, Vazquez J, Medina JF, et al. Analytical and clinical performance of the CDC real time RT-PCR assay for detection and typing of dengue virus. PLoS Negl Trop Dis. 2013;7(7):e2311.

3. Lanciotti RS, Kosoy OL, Laven JJ, Panella AJ, Velez JO, Lambert AJ, et al. Chikungunya virus in US travelers returning from India, 2006. Emerg Infect Dis. 2007;13(5):764-7.

4. Rances E, Ye YH, Woolfit M, McGraw EA, O'Neill SL. The relative importance of innate immune priming in Wolbachia-mediated dengue interference. PLoS Pathog. 2012;8(2):e1002548.

5. Warrilow D, Northill JA, Pyke A, Smith GA. Single rapid TaqMan fluorogenic probe based PCR assay that detects all four dengue serotypes. J Med Virol. 2002;66(4):524-8.

6. Santiago GA, Vazquez J, Courtney S, Matias KY, Andersen LE, Colon C, et al. Performance of the Trioplex real-time RT-PCR assay for detection of Zika, dengue, and chikungunya viruses. Nat Commun. 2018;9(1):1391.
