# Supplementary material for: Diagnostic performance of anti-Zika virus IgM, IgAM and IgG ELISAs during co-circulation of Zika, dengue, and chikungunya viruses in Brazil and Venezuela
Source: PLoS Negl Trop Dis. 2021 Apr 19;15(4):e0009336. doi: 10.1371/journal.pntd.0009336 (PMC8084345; doi:10.1371/journal.pntd.0009336)
Supplement: S2 Table — a Day of illness 1–5, shows the total number of acute samples tested per ELISA. Some patients have more than one acute sample tested (Table in S3 Table). b Day of illness 6 and over, shows the total number of follow-up samples tested per ELISA. Some patients have more than one follow-up sample tested (Table in S3 Table). c Number of acute samples that tested positive to the respective virus by RT-PCR and tested negative to all other viruses, thus co-infections are excluded. Neg = samples that tested negative to all three viruses and to DENV NS1. d Consists of samples positive for DENV by RT-PCR or samples positive by the DENV NS1 assay. Those positive by NS1 were either negative, indeterminate or not tested for DENV by RT-PCR. The number of samples NS1 positive per site were as follows: Rio de Janeiro and Resende, n = 9; Fortaleza and Recife, n = 8; Valencia, n = 12. e Includes one sample that tested DENV negative by RT-PCR but with an indeterminate result for DENV NS1. f Represents the number of total unique acute samples serologically tested per RT-PCR result and per study site. g Represents the number of total unique follow-up samples serologically tested per RT-PCR result and per study site. (DOCX) [file pntd.0009336.s002.docx]

|  |  |  |  | Number of acute^a^ samples | | |  | Number of follow-up^b^ samples | | |
| --- | --- | --- | --- | --- | --- | --- | --- | --- | --- | --- |
| Site | **Virus** | **Number Positive^c^** | **Total acute tested^f^** | **IgM** | **IgAM** | **IgG** | **Total follow-up tested^g^** | **IgM** | **IgAM** | **IgG** |
| Rio de Janeiro & Resende |  |  |  |  |  |  |  |  |  |  |
| N=109 | ZIKV | 15 | 17 | 17 | 17 | 17 | 11 | 11 | 11 | 11 |
|  | DENV^d^ | 22 | 25 | 21 | 25 | 25 | 16 | 16 | 16 | 16 |
|  | CHIKV | 19 | 26 | 25 | 26 | 25 | 7 | 7 | 7 | 7 |
|  | Neg | 50 | 56 | 56 | 56 | 56 | 40 | 40 | 40 | 40 |
| Recife & Fortaleza |  |  |  |  |  |  |  |  |  |  |
| N=434 | ZIKV | 32 | 50 | 39 | 50 | 39 | 15 | 14 | 15 | 13 |
|  | DENV^d^ | 76 | 98 | 94 | 93 | 98 | 44 | 42 | 44 | 43 |
|  | CHIKV | 134 | 100 | 9 | 100 | 100 | 100 | 9 | 100 | 100 |
|  | Neg | 165 | 175 | 164 | 162 | 164 | 107 | 104 | 103 | 103 |
| Valencia |  |  |  |  |  |  |  |  |  |  |
| N=364 | ZIKV^e^ | 30 | 29 | 20 | 29 | 29 | 24 | 11 | 24 | 1 |
|  | DENV^d^ | 38 | 40 | 0 | 40 | 37 | 28 | 0 | 28 | 0 |
|  | CHIKV | 121 | 121 | 0 | 121 | 120 | 0 | 0 | 0 | 0 |
|  | Neg | 91 | 95 | 2 | 95 | 90 | 70 | 2 | 70 | 1 |
| Total no. of ELISAs performed |  |  | **832** | **447** | **814** | **800** | **462** | **256** | **458** | **335** |
|  |  |  |  | *2,061* | | |  | *1,049* | | |
