# Supplementary material for: Diagnostic performance of anti-Zika virus IgM, IgAM and IgG ELISAs during co-circulation of Zika, dengue, and chikungunya viruses in Brazil and Venezuela
Source: PLoS Negl Trop Dis. 2021 Apr 19;15(4):e0009336. doi: 10.1371/journal.pntd.0009336 (PMC8084345; doi:10.1371/journal.pntd.0009336)
Supplement: S3 Table — a Brazil: Recife, Fortaleza, Rio de Janeiro, and Resende; Venezuela: Valencia. Neg = samples that tested negative to all ZIKV, DENV, CHIKV by RT-PCR and negative to DENV NS1 antigen by the Platelia NS1 assay. NA, not applicable. Indicates that no samples were tested for the respective ELISA. (DOCX) [file pntd.0009336.s003.docx]

| **Virus/antigen** | **Study site(s)^a^** | **Acute samples** | | | **Follow-up samples** | | |
| --- | --- | --- | --- | --- | --- | --- | --- |
|  |  | **IgM** | **IgAM** | **IgG** | **IgM** | **IgAM** | **IgG** |
| ZIKV+ | Rio de Janeiro and Resende | 2 | 2 | 2 | 0 | 0 | 0 |
|  | Recife and Fortaleza | 8 | 18 | 7 | 1 | 2 | 0 |
|  | Valencia | 0 | 0 | 0 | 0 | 0 | 0 |
| DENV+/NS1+ | Rio de Janeiro and Resende | 3 | 3 | 3 | 0 | 0 | 0 |
|  | Recife and Fortaleza | 20 | 19 | 24 | 0 | 1 | 0 |
|  | Valencia | NA | 2 | 0 | NA | 0 | NA |
| CHIKV+ | Rio de Janeiro and Resende | 6 | 7 | 6 | 0 | 0 | 0 |
|  | Recife and Fortaleza | 0 | 0 | 0 | 0 | 0 | 0 |
|  | Valencia | NA | 0 | 0 | NA | NA | NA |
| Neg | Rio de Janeiro and Resende | 6 | 6 | 6 | 0 | 0 | 0 |
|  | Recife and Fortaleza | 34 | 32 | 34 | 5 | 3 | 3 |
|  | Valencia | 0 | 5 | 0 | 0 | 0 | 0 |
