# Supplementary material for: Diagnostic performance of anti-Zika virus IgM, IgAM and IgG ELISAs during co-circulation of Zika, dengue, and chikungunya viruses in Brazil and Venezuela
Source: PLoS Negl Trop Dis. 2021 Apr 19;15(4):e0009336. doi: 10.1371/journal.pntd.0009336 (PMC8084345; doi:10.1371/journal.pntd.0009336)
Supplement: S4 Table — N represents the number of patients with a sample that was tested for the corresponding immunoassay; some patients have both acute and follow-up samples: IgM, n = 34; IgAM, n = 34. (DOCX) [file pntd.0009336.s004.docx]

| **ZIKV RT-PCR+**  Number of patients=77 | | | | | | | | | | |
| --- | --- | --- | --- | --- | --- | --- | --- | --- | --- | --- |
| **Serological test** | **Acute (day 1-5)** | | | | | **Follow-up (≥ 6 day ≤ 31)** | | | | |
|  | N | Pos | Neg | Ind | % Sensitivity (95 % CI) | N | Pos | Neg | Ind | % Sensitivity  (95 % CI) |
| **IgM** | 66 | 6 | 58 | 2 | 9.4 (3.9-20.0) | 35 | 10 | 23 | 2 | 30.3 (16.2-48.9) |
| **IgAM** | 66 | 8 | 54 | 4 | 12.9 (6.1-24.4) | 35 | 32 | 2 | 1 | 94.1 (78.9-99.0) |
