# Supplementary material for: Diagnostic performance of anti-Zika virus IgM, IgAM and IgG ELISAs during co-circulation of Zika, dengue, and chikungunya viruses in Brazil and Venezuela
Source: PLoS Negl Trop Dis. 2021 Apr 19;15(4):e0009336. doi: 10.1371/journal.pntd.0009336 (PMC8084345; doi:10.1371/journal.pntd.0009336)
Supplement: S5 Table — OR, Odds ratio; CI, confidence interval. The table shows the OR and the respective 95% CIs for the association between testing anti-ZIKV IgG positive and key factors at study enrollment. Results are shown for the univariable and multivariable models, followed by a stepwise regression analysis. Prior to adjustment, we found statistically significant associations between testing anti-ZIKV IgG positive and having fever, being anti-DENV IgG positive at enrollment, being CHIKV RT-PCR positive, being DENV RT-PCR positive or NS1 positive, year, age, and study site (Recife and Valencia compared to Rio de Janeiro/Resende). In the multivariable model, the magnitude of the associations for age, year, and being DENV RT-PCR positive/NS1 positive slightly decreased but remained statistically significant. In contrast, the magnitude of the associations for fever, testing DENV IgG positive at enrollment, and being CHIKV RT-PCR positive increased and remained statistically significant for all. Study site was excluded from the multivariable regression analysis due to collinearity with the variable ‘DENV IgG+.’ Stepwise regression analysis revealed that being DENV IgG positive at enrollment and being CHIKV RT-PCR positive significantly increased the odds of testing ZIKV IgG positive at enrollment by 5.6- and 3.3-fold, respectively. This may be the result of antibody cross-reactivity—in the case of dengue—or due to the overlapping arbovirus epidemics that preceded (CHIKV before ZIKV in Venezuela) or followed the ZIKV epidemic (CHIKV following the ZIKV epidemic in Brazil) (S3 Fig). Having fever decreased the odds (OR = 0.51, CI: 0.33–0.77, p = 0.002) of testing ZIKV IgG positive at enrollment, likely because fever is a marker of acute infection and not past flavivirus exposure. We also found that having tested DENV RT-PCR positive/NS1 positive significantly decreased the odds (OR = 0.32, CI: 0.15–0.62, p = 0.001) of testing ZIKV IgG positive. Being ZIKV IgG positive was significantly mor [file pntd.0009336.s005.docx]

| Factors | ZIKV IgG –  n (%) | ZIKV IgG+  n (%) | OR (95% CI, p-value)  univariable | OR (95% CI, p-value)  multivariable | OR (95% CI, p-value)  stepwise |
| --- | --- | --- | --- | --- | --- |
| *Fever* |  |  |  |  |  |
| No | 144 (37.2) | 97 (46.4) | 1.0 | 1.0 | 1.0 |
| Yes | 243 (62.8) | 112 (53.6) | 0.68 (0.49-0.96, p=0.029) | 0.51 (0.33-0.78, p=0.002) | 0.51 (0.33-0.77, p=0.002) |
| *Skin flush* |  |  |  |  |  |
| No | 295 (76.2) | 163 (78.0) | 1.0 | 1.0 | 1.0 |
| Yes | 92 (23.8) | 46 (22.0) | 0.90 (0.60-1.35, p=0.626) | 0.65 (0.39-1.10, p=0.110) | 0.69 (0.43-1.10, p=0.119) |
| *Skin rash* |  |  |  |  |  |
| No | 258 (66.7) | 131 (62.7) | 1.0 | 1.0 | - |
| Yes | 129 (33.3) | 78 (37.3) | 1.19 (0.84-1.69, p=0.329) | 1.12 (0.69-1.81, p=0.659) | - |
| *DENV IgG+* |  |  |  |  |  |
| No | 320 (82.7) | 97 (46.4) | 1.0 | 1.0 | 1.0 |
| Yes | 67 (17.3) | 112 (53.6) | 5.51 (3.79-8.09, p<0.001) | 5.72 (3.70-8.96, p<0.001) | 5.63 (3.66-8.78, p<0.001) |
| *Study site* |  |  |  |  |  |
| Rio de Janeiro / Resende | 79 (20.4) | 17 (8.1) | 1.0 | - | - |
| Fortaleza | 89 (23.0) | 19 (9.1) | 0.99 (0.48-2.06, p=0.983) | - | - |
| Recife | 66 (17.1) | 104 (49.8) | 7.32 (4.07-13.80, p<0.001) | - | - |
| Valencia | 153 (39.5) | 69 (33.0) | 2.10 (1.18-3.90, p=0.015) | - | - |
| *Year* |  |  |  |  |  |
| 2012-14 | 151 (39.0) | 46 (22.0) | 1.0 | 1.0 | 1.0 |
| 2015-16 | 236 (61.0) | 163 (78.0) | 2.27 (1.55-3.36, p<0.001) | 1.83 (1.12-3.01, p=0.016) | 1.86 (1.15-3.04, p=0.012) |
| *Age* |  |  |  |  |  |
| 5-15 | 121 (31.3) | 24 (11.5) | 1.0 | 1.0 | 1.0 |
| 16+ | 266 (68.7) | 185 (88.5) | 3.51 (2.21-5.76, p<0.001) | 2.03 (1.20-3.53, p=0.010) | 2.02 (1.19-3.52, p=0.010) |
| *Sex* |  |  |  |  |  |
| Female | 200 (51.7) | 115 (55.0) | 1.0 | 1.0 | - |
| Male | 187 (48.3) | 94 (45.0) | 0.87 (0.62-1.22, p=0.435) | 1.05 (0.70-1.57, p=0.811) | - |
| *RT-PCR* |  |  |  |  |  |
| RT-PCR Neg | 149 (38.5) | 82 (39.2) | 1.0 | 1.0 | 1.0 |
| CHIKV RT-PCR + | 93 (24.0) | 88 (42.1) | 1.72 (1.16-2.56, p=0.007) | 3.18 (1.94-5.32, p<0.001) | 3.25 (1.99-5.39, p<0.001) |
| DENV RT-PCR + / NS1 + | 105 (27.1) | 13 (6.2) | 0.22 (0.11-0.41, p<0.001) | 0.32 (0.15-0.62, p=0.001) | 0.32 (0.15-0.62, p=0.001) |
| ZIKV RT-PCR + | 40 (10.3) | 26 (12.4) | 1.18 (0.67-2.06, p=0.562) | 0.80 (0.41-1.56, p=0.522) | 0.83 (0.43-1.58, p=0.569) |
