# Supplementary material for: Diagnostic performance of anti-Zika virus IgM, IgAM and IgG ELISAs during co-circulation of Zika, dengue, and chikungunya viruses in Brazil and Venezuela
Source: PLoS Negl Trop Dis. 2021 Apr 19;15(4):e0009336. doi: 10.1371/journal.pntd.0009336 (PMC8084345; doi:10.1371/journal.pntd.0009336)
Supplement: S6 Table — N represents the number of patients with samples; some patients have both acute and follow-up samples: IgM, n = 56; IgAM, n = 56. (DOCX) [file pntd.0009336.s006.docx]

| **DENV RT-PCR+ /NS1+**  Number of patients=136 | | | | | | | | | | |
| --- | --- | --- | --- | --- | --- | --- | --- | --- | --- | --- |
| **Serological test** | **Acute (day 1-5)** | | | | | **Follow-up (≥ 6 day ≤ 31)** | | | | |
|  | N | Pos | Neg | Ind | % Specificity  (95 % CI) | N | Pos | Neg | Ind | % Specificity  (95 % CI) |
| **IgM** | 92 | 2 | 89 | 1 | 97.8 (91.5-99.6) | 57 | 4 | 52 | 1 | 92.9 (81.9-97.7) |
| **IgAM** | 92 | 6 | 83 | 3 | 93.3 (85.4-97.2) | 57 | 10 | 44 | 3 | 81.5 (68.1-90.3) |
