# Supplementary figures and images for: Diagnostic performance of anti-Zika virus IgM, IgAM and IgG ELISAs during co-circulation of Zika, dengue, and chikungunya viruses in Brazil and Venezuela
Source: PLoS Negl Trop Dis. 2021 Apr 19;15(4):e0009336. doi: 10.1371/journal.pntd.0009336 (PMC8084345; doi:10.1371/journal.pntd.0009336)

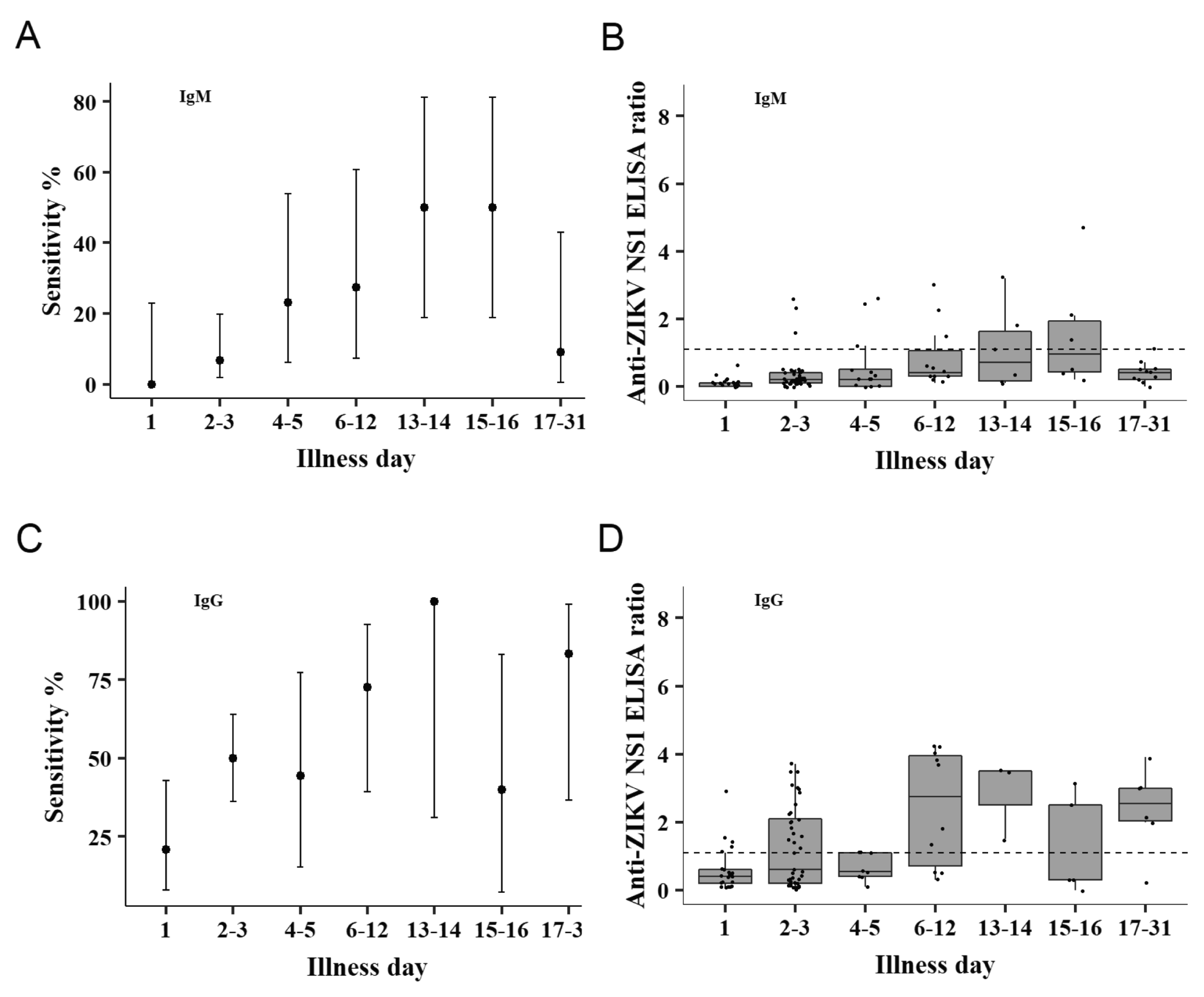

Supplement: S1 Fig — (A) Sensitivity of IgM to ZIKV per illness day. IgM immunoassay results from 108 samples collected between day 1 and 31 from ZIKV positive patients were used to calculate assay sensitivity at different illness days. The number of tests corresponding to each day or day range are the following: day 1, n = 17; days 2–3, n = 44; days 4–5, n = 13; days 6–12, n = 11; days 13–14, n = 6; days 15–16, n = 6 and days 17–31, n = 11. Bars represent the 95% confidence intervals. (B) Box plot of anti-ZIKV IgM ratio values per illness day. The number of tests corresponding to each day or day range are the same as in (A). The dashed horizontal line indicates the assay’s cut-off ratio value for positive results set at 1.1. (C) Sensitivity of IgG to ZIKV per illness day. IgG immunoassay results from 104 samples collected between day 1 and 31 from ZIKV positive patients were used to calculate assay sensitivity at different illness days. The number of tests corresponding to each day or day range are the following: day 1, n = 24; days 2–3, n = 46; days 4–5, n = 9; days 6–12, n = 11; days 13–14, n = 3; days 15–16, n = 5 and days 17–31, n = 6. Bars represent the 95% confidence intervals. (D) Box plot of anti-ZIKV IgG ratio values per illness day. The number of tests corresponding to each day or day range differ slightly from (C) since some antibody reference values were not available: day 1, n = 21; days 2–3, n = 41; days 4–5, n = 8; days 6–12, n = 10; days 13–14, n = 3; days 15–16, n = 5 and days 17–31, n = 6. The dashed horizontal line indicates the assay’s cut-off ratio value for positive results set at 1.1. (TIF) [file pntd.0009336.s007.tif]

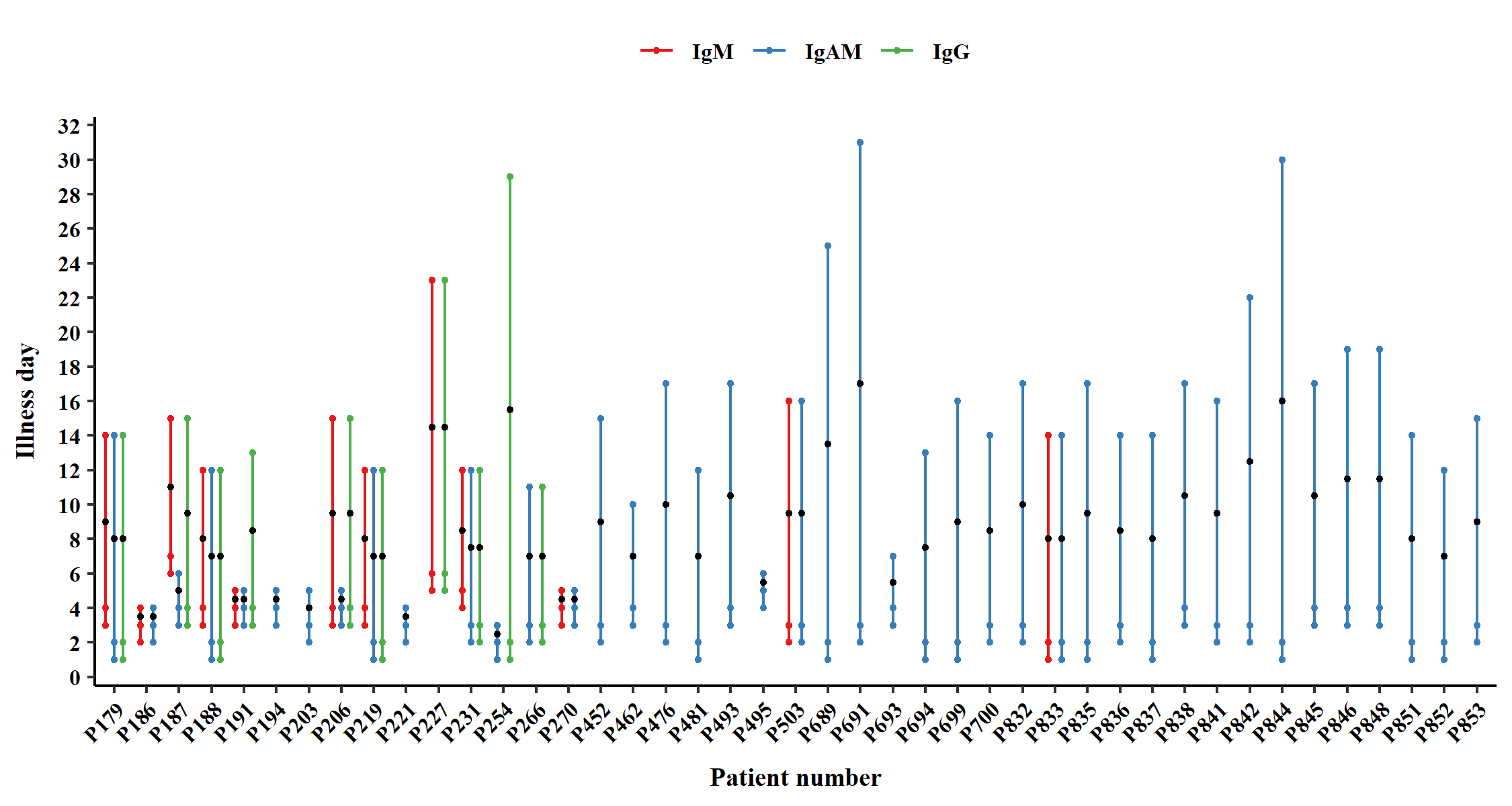

Supplement: S2 Fig — IgM (red line), IgAM (blue line), IgG (green line). The timeframe for seroconversion detection is shown for each patient that had an initial sample that tested negative to one or more of the antibody ELISAs and who seroconverted at a later timepoint. The colored dots closest to the x-axis indicate the illness day at which the first sample was collected and tested negative for each immunoassay. The second colored dot from bottom to top, indicates the first illness day at which seroconversion could have occurred, which corresponds to one day after the first sample tested negative. The topmost colored dot indicates the illness day at seroconversion detection. Black dots indicate the median illness day. (TIF) [file pntd.0009336.s008.tif]

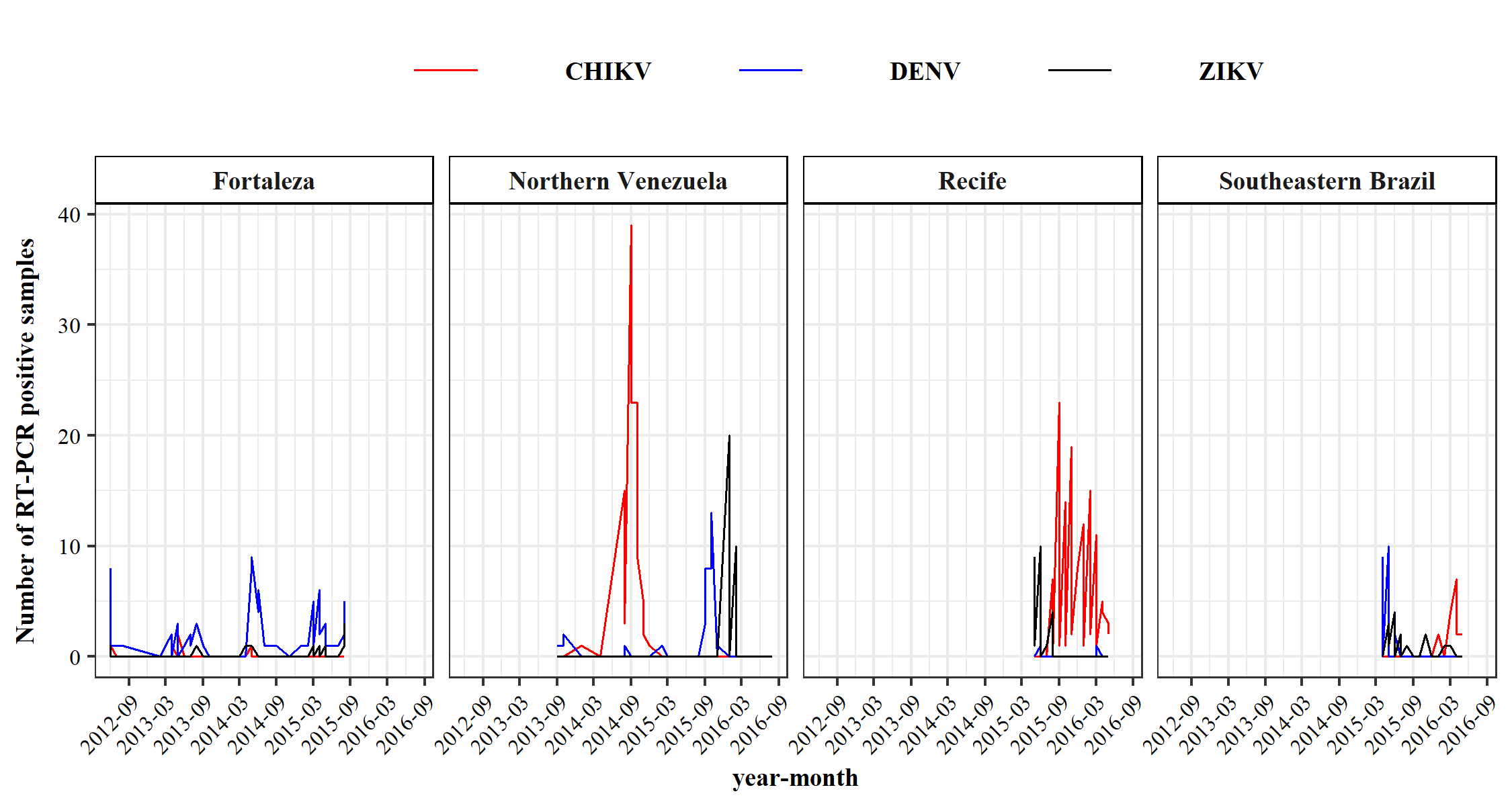

Supplement: S3 Fig — ZIKV (gray line), DENV (blue line), CHIKV (red line). Epidemiological curves are shown for Fortaleza and Recife in Northeastern Brazil, Rio de Janeiro and Resende in Southeastern Brazil, and Valencia in Northern Venezuela. (TIF) [file pntd.0009336.s009.tif]
